# Kant’s Theory of Radical Evil and its Franciscan Forebears

**DOI:** 10.1515/nzsth-2023-0022

**Published:** 2023-08-31

**Authors:** Lydia Schumacher

**Affiliations:** Reader in Historical and Philosophical Theology, King’s College London, London, United Kingdom of Great Britain and Northern Ireland

**Keywords:** evil, Kant, Franciscans, free will, *Summa Halensis*, das Böse, Kant, Franziskaner, freier Wille, *Summa Halensis*

## Abstract

This article argues that Kant’s famous theory of ‘radical evil’, according to which there is a natural propensity for evil as well as good in all human beings, has precedent in the medieval Franciscan intellectual tradition. In the early thirteenth century, members of this tradition, inspired by its founder Alexander of Hales, developed a novel account of free will, according to which the will is capable of choosing between equally legitimate options of good and evil. In affirming this, early Franciscans departed from the longstanding tradition of Augustine, for whom free will can only choose the good, since evil is merely a privation of the good that limits human freedom. By the same token, they anticipated the Kantian contention that freedom entails the ability to choose between good and evil maxims.

## Zusammenfassung

Dieser Artikel argumentiert, dass Kants berühmte Theorie vom „radikalen Bösen“, gemäß der es eine natürliche Anlage zum Bösen und zum Guten in allen Menschen gibt, Vorläufer in der mittelalterlichen franziskanischen intellektuellen Tradition hat. Im frühen 13. Jahrhundert entwickelten Mitglieder dieser Tradition, inspiriert von ihrem Gründer Alexander von Hales, eine neue Vorstellung vom freien Willen, gemäß der der Wille in der Lage ist, zwischen gleichermaßen legitimen Optionen des Guten und des Bösen zu wählen. Damit wichen die frühen Franziskaner von der langjährigen Tradition des Augustinus ab, für den der freie Wille nur das Gute wählen kann, da das Böse lediglich eine Mangelerscheinung des Guten ist, die die menschliche Freiheit einschränkt. Gleichzeitig antizipierten sie die kantische Behauptung, dass Freiheit die Fähigkeit beinhaltet, zwischen guten und bösen Maximen zu wählen.

In his *Religion within the Boundaries of Bare Reason* (1973), Immanuel Kant (1724–1804) famously argued that there is an innate or natural propensity for evil in all human beings. He described this propensity as ‘radical’, from the Latin word *radix,* which means ‘root’, because human beings possess it as the ‘subjective basis for the possibility of an inclination’1Immanuel Kant, *Die Religion innerhalb der Grenzen der bloßen Vernunft,* 6.29, trans. Werner S. Pluhar and Stephen R. Palmquist, *Religion within the Bounds of Bare Reason* (London: Hackett Publishing Company, 2009, 31. Here and in what follows, all references to Kant’s writings will be to: *Kant’s Gesammelte SchriftenAkademieausgabe*, Königlich Preußische Akademie der Wissenschaften (Berlin: Reimer; later: de Gruyter), 1900 ff. towards evil.2Kant, *Religion,* 6.32, ‘So werden wir diesen einen natürlichen Hang zum Bösen, und da er doch immer selbstverschuldet sein muß, ihn selbst ein radicales, angebornes, (nichts destoweniger aber uns von uns selbst zugezogenes) Böse in der menschlichen Natur nennen können,’ trans. Pluhar/Palmquist, 35: ‘Presumably, therefore, we may call this basis a natural propensity to evil, and, since it must yet always be something of which one is oneself guilty, we may even call it a radical, innate evil in human nature.’ More specifically, Kant contends that the propensity to radical evil is latent in the power of free will (*freie Wille*), which is the faculty on account of which humans can choose between what Kant calls good or evil ‘maxims’. As Kant writes:

The basis of evil cannot lie in any object *determining* the power of choice through inclination, not in any natural impulse, but can lie only in a rule that the power of choice itself – for the use of its freedom – makes for itself, i. e., in a maxim... Thus when we say, the human being is by nature good, or, he is by nature evil, this means no more than this: he contains a first basis (inscrutable to us) for the adoption of good maxims or the adoption of evil (unlawful) ones,’ i. e. free will.3Kant, *Religion,* 6.21, ‘Mithin kann in keinem die Willkür durch Neigung bestimmenden Objecte, in keinem Naturtriebe, sondern nur in einer Regel, die die Willkür sich selbst für den Gebrauch ihrer Freiheit macht, d. i. in einer Maxime, der Grund des Bösen liegen... Wenn wir also sagen: der Mensch ist von Natur gut, oder: er ist von Natur böse, so bedeutet dieses nur so viel als: er enthält einen (uns unerforschlichen) ersten Grund der Annehmung guter, oder der Annehmung böser (gesetzwidriger) Maximen,’ trans. Pluhar/Palmquist, 21.

Since its initial formulation, Kant’s theory of evil has been regarded, with good reason, as a landmark in the history of philosophy, which spelled the ultimate demise of the Augustinian theory of evil as a ‘privation’ or absence of the good that had remained popular throughout the Middle Ages. In this article, however, I seek to demonstrate that a theory similar to Kant’s was already developed in the Middle Ages themselves, paradoxically, within a school of thought, belonging to the Franciscan religious order, which at least in the first years of its existence, was regarded as the last bastion of Augustinianism. As I will show below, the founders of this school, including Alexander of Hales and John of La Rochelle, who worked in the early years following the establishment of the first chartered university at Paris (1200), reinterpreted Augustine and his great medieval follower Anselm on evil in a way that effectively subverted privation theory.

Additionally, early Franciscans invoked more recently recovered authorities like John of Damascus, in order to advance the contention that evil is an intrinsic possibility for human nature, which is rooted in free will. To demonstrate this, I will outline the contours of Augustine’s view of evil and its elaboration by Anselm. On the account of these thinkers, as noted, evil is not a substantial option for free will as Kant supposed, let alone one that is rooted in human nature. Rather, it consists in an absence or lack of the good which results from the will’s failure to prefer greater over lesser goods. This was the paradigm the Franciscans fundamentally reoriented in a manner that anticipated Kant.

In recent years, a number of scholars such as Ludger Honnefelder have noted certain affinities between Kant and Franciscan scholastics, especially Duns Scotus, who was himself educated on works written by Alexander of Hales and John of La Rochelle*.* According to Honnefelder, the ideas of Franciscan scholastics likely reached Kant not directly but by means of his contemporaries such as Wolff and Baumgarten, who were avid readers not only of Scotus but also of Francisco Suarez, a key channel of Scotist thought to early modernity. Suarez apparently affirmed a role for positive as well as privative evils, which Pini claims that Scotus continued to affirm.4Giorgio Pini, ‘Scotus,’ in *A History of Evil in the Middle Ages, 450–1450 CE,* ed. Andrew Pinsent (*The History of Evil*, vol. 2) (London: Routledge, 2018), 213–25. Jorge J. E. Gracia, ‘Evil and the Transcendentality of Goodness: Suárez’s Solution to the Problem of Positive Evils,’ in *Being and Goodness: The Concept of the Good in Metaphysics and Philosophical Theology*, ed. Scott Macdonald (New York: Cornell University Press, 1990), 151–76.

As Newlands has shown, other early modern thinkers like Descartes and Leibniz likewise offered revisionist accounts of privation theory, which Spinoza ultimately rejected.5Samuel Newlands, ‘Evils, Privations and the Early Moderns,’ in *Evil: A History,* ed. Andrew Chignell (Oxford: Oxford University Press, 2019), 273–305 Thus, the popularity of the theory clearly waned in early modernity. Another possible source of Kant’s criticism of privation theory was his pietist tradition, which followed Luther in offering a more dualist account of good as opposed to evil.6Dietmar Wyrwa, ‘Augustin und Luther über das Böse,’ *Philotheos* 3 (2003), 154–75. Although it lies beyond the scope of my current project to trace the precise lines through which the early Franciscan view could have reached Kant, the affinities between the two sources can be established on the basis of the account of Kant’s theory of evil that I offer below.7In a study of the theory of the transcendentals, Ludger Honnefelder has traced a line of development from Scotus through Suarez to Kant. *Scientia transcendens. Die formale Bestimmung der Seiendheit in der Metaphysik des Mittelalters und der Neuzeit (Duns Scotus-Suárez-Wolff-Kant-Peirce)* (Hamburg: Meiner, 1992). See also Honnefelder’s summary article, ‘Metaphysics as a Discipline: From the Transcendental Philosophy of the Ancients to Kant’s Notion of Transcendental Philosophy,’ in *The Medieval Heritage in Early Modern Metaphysics and Modal Theory 1400–1700,* ed. R. L. Friedman and L. O. Nielsen (Kluwer Academic Publishers, 2003), 53–74.

## Kant on Radical Evil

Kant’s account of radical evil is constructed on the foundation of the ethical views he presents in his *Critique of Practical Reason* and more briefly in his *Groundwork of the Metaphysics of Morals.* There he outlines his deontological (duty) ethic according to which any action is moral if and only if it conforms to the moral law. Thus, Kant writes that our main obligation as humans is to ‘act as if the maxim of your action were to become by your will a universal law.’8Immanuel Kant, *Grundlegung der Metaphysik der Sitten,* 4:421, ‘Handle so, als ob die Maxime deiner Handlung durch deinen Willen zum allgemeinen Naturgesetze werden sollte,’ ed./trans. Mary Gregor and Jens Zimmerman, in *Groundwork of the Metaphysics of Morals,* (Cambridge: Cambridge University Press, 2011), 34. In this regard, Kant insists that we must treat all persons, including the self, as ends-in-themselves rather than as means to achieving some other end.9Kant, *Grundlegung,* 4:428, ‘Nun sage ich: der Mensch und überhaupt jedes vernünftige Wesen existirt als Zweck an sich selbst, nicht bloß als Mittel zum beliebigen Gebrauche für diesen oder jenen Willen, sondern muß in allen seinen sowohl Gebrauche für diesen oder jenen Willen, sondern muß in allen seinen sowohl auf sich selbst, als auch auf andere vernünftige Wesen gerichteten Handlungen jederzeit zugleich als Zweck betrachtet werden,’ ed./trans. Gregor/Zimmerman, 40: ‘Now I say: a human being and generally every rational being exists as an end in itself, not merely as a means for the discretionary use for this or that will, but must in all its actions, whether directed towards itself or also to other rational beings, always be considered at the same time as an end.’ He gives several examples of how the maxim can be applied in practice.

In the first, an individual contemplates suicide in order to alleviate their personal suffering. Kant notes that the individual in this case treats themselves as a means to relieving suffering rather than as an end. The maxim of their action is therefore subjective, geared towards achieving personal relief and happiness. As such, this maxim fails to provide an objective and rational principle that is suited to serving as a moral basis for all persons at all places and at all times. On these grounds, Kant insists that the maxim of the individual’s action cannot be turned into a universally applicable moral law. The same conclusion is reached in the next example Kant cites of a person who considers asking to borrow money from a friend but has no intention of repaying it. Here, Kant shows that the person treats their friend as a means to personal benefit rather than as an end in itself.

Thus, the maxim of this action is subjective, geared towards personal happiness, rather than rooted in reason and objectivity. More specifically, Kant describes the action in terms of self-love, which is allowed in the case of an evil maxim to take priority over the duty to the moral law. In his *Religion,* Kant elaborates on the nature of self-love, by distinguishing between three different human predispositions: animality, humanity, and personality. The animal aspect of human nature involves a purely physical self-love that has no basis in reason; it is orientated towards self-preservation, procreation, and society or community with others.10Kant, *Religion,* 6.26, trans. Pluhar/Palmquist, 28. Humanity is self-love that concerns the desire to be happy in comparison to others – to gain recognition from and equality with others and not to be inferior to them.11Kant, *Religion,* 6.27, ‘Die Anlagen für die Menschheit können auf den allgemeinen Titel der zwar physischen, aber doch vergleichenden Selbstliebe (wozu Vernunft erfordert wird) gebracht werden: sich nämlich nur in Vergleichung mit andern als glücklich oder unglücklich zu beurtheilen. Von ihr rührt die Neigung her, sich in der Meinung anderer einen Werth zu verschaffen; und zwar ursprünglich bloß den der Gleichheit: keinem über sich Überlegenheit zu verstatten, mit einer beständigen Besorgniß verbunden, daß andere darnach streben möchten; woraus nachgerade eine ungerechte Begierde entspringt, sie sich über Andere zu erwerben,’ trans. Pluhar/Palmquist, 29: ‘The predispositions to humanity can be brought under the general title of a no doubt physical but yet comparing self-love (for which reason is required): namely to judge oneself happy or unhappy only by comparison with others. From this self-love stems the inclination to procure a worth for oneself in the opinion of others, originally, to be sure, merely that of equality: to permit no one superiority over oneself, combined with a constant worry that others might strive for this, from which arises gradually an unjust desire to gain superiority over others.’

This desire is rooted in practical reason but is nonetheless subservient to incentives or objectives other than the moral law. By contrast, personality consists in the power of free choice that seeks to observe the moral law for its own sake.12Kant, *Religion,* 6.27, ‘Die Anlage für die Persönlichkeit ist die Empfänglichkeit der Achtung für das moralische Gesetz, als einer für sich hinreichenden Triebfeder der Willkür. Die Empfänglichkeit der bloßen Achtung für das moralische Gesetz in uns wäre das moralische Gefühl, welches für sich noch nicht einen Zweck der Naturanlage ausmacht, sondern nur sofern es Triebfeder der Willkür ist. Da dieses nun lediglich dadurch möglich wird, daß die freie Willkür es in ihre Maxime aufnimmt: so ist Beschaffenheit einer solchen Willkür der gute Charakter,’ trans. Pluhar/Palmquist, 30: ‘The predisposition to personality is the receptivity to respect for the moral law, as an incentive, sufficient by itself, of the power of choice. This receptivity to mere respect for the moral law within us would be the moral feeling, which by itself does not yet amount to a purpose of the natural predisposition, but amounts to such a purpose only insofar as it is an incentive of the power of choice. Now, since this becomes possible solely through the free power of choice’s admitting the moral feeling into its maxim, the constitution of such a power of choice is a good character.’ As such, it is fully objective, rooted in practical reason, possessing no other goal than to observe the moral law. As Kant elaborates, the first two predispositions of human being – animality and humanity – which are guided by self-love, do not necessarily conflict with the moral law and can actually facilitate its observance. The important point for Kant is that such subjective considerations should not be allowed to trump the objective duty to the moral law. Thus, Kant writes:

All these predispositions in the human being are not only (negatively) good (they do not conflict with the moral law) but are also predispositions to the good (they further compliance with that law). They are original; for they belong to the possibility of human nature. The human being can indeed use the first two contra-purposively, but cannot extirpate either of them.13Kant, *Religion,* 6.28, ‘Alle diese Anlagen im Menschen sind nicht allein (negativ) gut (sie widerstreiten nicht dem moralischen Gesetze), sondern sind auch Anlagen zum Guten (sie befördern die Befolgung desselben). Sie sind ursprünglich; denn sie gehören zur Möglichkeit der menschlichen Natur. Der Mensch kann die zwei ersteren zwar zweckwidrig brauchen, aber keine derselben vertilgen,’ trans. Pluhar/Palmquist, 30.

As Kant elaborates, there are three levels at which human self-love can be allowed to supersede the duty to the moral law and thus produce an evil maxim rather than a good one. These are frailty, impurity, and depravity.14Kant, *Religion,* 6.29, ‘Man kann sich drei verschiedene Stufen desselben denken. Erstlich ist es die Schwäche des menschlichen Herzens in Befolgung genommener Maximen überhaupt, oder die Gebrechlichkeit der menschlichen Natur; zweitens der Hang zur Vermischung unmoralischer Triebfedern mit den moralischen (selbst wenn es in guter Absicht und unter Maximen des Guten geschähe), d. i. die Unlauterkeit; drittens der Hang zur Annehmung böser Maximen, d. i. die Bösartigkeit der menschlichen Natur, oder des menschlichen Herzens,’ trans. Pluhar/Palmquist, 32: ‘One can think of three different levels of this propensity. It is first, the human heart’s weakness as such in complying with adopted maxims, or the frailty of human nature; second, the propensity to mix immoral incentives with the moral ones (even if this were done with good intention and under maxims of the good), i. e., impurity; third, the propensity to adopt evil maxims, i. e., the wickedness of human nature, or of the human heart.’ The first involves a weakness of the will to comply with the maxims of the moral law. In the second case, actions which abide by the duty to obey the moral law are not done from duty. Thus, there is a conformity to the law in deed but not in spirit, which makes the action impure. In the last case of depravity, we prefer our own desires over obedience to the moral law without compunction. This inverts the proper order of the maxims – to obtain happiness and to observe the moral law – subjecting the latter to the former rather than the other way around.15Kant, *Religion,* 6.36–37, trans. Pluhar/Palmquist, 40.

Here, Kant clearly criticises the sort of Aristotelian virtue ethic that is orientated towards achieving happiness (*eudaimonia*), which in Aristotelian terms refers more precisely to human excellence or flourishing than to happiness as such.16Manfred Kuehn stresses the differences between Aristotle and Kant in, ‘Kant and Aristotle on Ethics,’ in *The Reception of Aristotle’s Ethics,* ed. Jon Miller (Cambridge: Cambridge University Press, 2013), 244–61. As we have seen, the pursuit of happiness cannot provide an objective basis for moral action in Kant’s view, as it is prone to indulging potentially selfish self-love. In this regard, Kant also takes issue with the Aristotelian notion that moral action is a matter of discretion concerning what is right to do in particular circumstances.17Aristotle, *Nicomachean Ethics,* ed. Roger Crisp (Cambridge: Cambridge University Press, 2004), 2.9, 35: ‘So too anyone can get angry, or give and spend money – these are easy; but doing them in relation to the right person, in the right amount, at the right time, with the right aim in view, and in the right way – that is not something anyone can do, nor is it easy. This is why excellence in these things is rare, praiseworthy and noble.’ As Aristotle writes:

Matters concerned with conduct and questions of what is good for us have no fixity, any more than matters of health. The general account being of this nature, the account of particular cases is yet more lacking in exactness; for they do not fall under any art or precept but the agents themselves must in each case consider what is appropriate to the occasion, as happens also in the art of medicine or of navigation.18Aristotle, *Nicomachean Ethics,* 2.2, ed. Crisp, 24–25.

The lack of ‘fixity’ in ethical matters of which Aristotle speaks in this context was clearly incompatible with Kant’s concern to find a universally applicable, objective moral law. While Kant does not see virtue as the *basis* for moral virtue, he nevertheless affirms that virtue is the consequence of following the moral law and indeed of perfecting the art of doing so.19There has been considerable debate in recent years on whether and the extent to which Kant endorses virtue theory as well as his rejection or adaption of Aristotle’s virtue theory. See Anne Margaret Baxley, *Kant’s Theory of Virtue: The Value of Autocracy* (Cambridge: Cambridge University Press, 2010). Nancy Sherman, *Making a Necessity of Virtue: Aristotle and Kant on Virtue* (Cambridge University Press, 1997). Monica Betzler (ed.), *Kant’s Ethics of Virtue* (New York: Walter de Gruyter, 2008). As Kant writes: ‘When the firm resolve in complying with one’s duty has become a proficiency, it is also called virtue.’20Kant, *Religion,* 6.46–47, trans. Pluhar/Palmquist, 53. See Lara Denis, ‘Kant’s Conception of Virtue,’ in *The Cambridge Companion to Kant and Modern Philosophy,* ed. Paul Guyer (Cambridge: Cambridge University Press, 2006), 510 in 505–37. Interestingly, Kant follows Aristotle further in describing virtue as a matter of the will’s habituation, not in striking the mean between excess and deficiency, as Aristotle supposed, but simply in observing the moral law:

Hence virtue in this sense is acquired little by little and means to some a long habituation (in observing the law), whereby the human being, through gradual reforms of his conduct and stabilization of his maxims, has passed over from the propensity to vice to an opposite propensity.21Adam Cureton and Thomas E. Hill, ‘Kant on Virtue and the Virtues,’ in *Cultivating Virtue*, ed. Nancy Snow (Oxford: Oxford University Press, 2014), 87–110.

As noted above, the main difference between Aristotle and Kant on this score is that the former saw habituation as a matter of cultivating those qualities or virtues that allow for spontaneous moral judgments, while the latter understood it in terms of the consistent application of the moral law. For Kant, in sum, virtue is ‘the moral strength of a man’s will in fulfilling his duty.’22Immanuel Kant, *The Metaphysics of Morals,* ed. Mary Gregor (Cambridge: Cambridge University Press, 1991), 206. Thus, there are resonances of Aristotle in Kant, despite his rejection of eudaimonism. These resonances come into further relief in Kant’s account of the ‘degrees’ in the propensity to evil, which strongly correlate to the degrees of virtue and vice of which Aristotle speaks in *Nicomachean Ethics*, Book 7.

The first degree of virtue is temperance, which involves wanting to do what is right for its own sake, or out of a conviction that this is in one’s best interests, which seems to correspond to Kant’s ideal of a moral person.23Aristotle, *Nicomachean Ethics,* 2.4, ed. Crisp, 27–28: ‘But actions done in accordance with virtues are done in a just or temperate way not merely by having some quality of their own, but rather if the agent acts in a certain state, namely, first, with knowledge, secondly, from rational choice, and rational choice of the actions for their own sake, and, thirdly, from a firm and unshakeable character.’ The second degree of continence entails wanting to do something evil but stopping oneself from doing it and corresponds to Kant’s category of impurity. Finally, incontinence involves knowing an act is wrong but doing it anyways out of a weakness of will (*akrasia*) to be virtuous, a notion which corresponds to Kant’s frailty. The final stage for Aristotle is simply that of vice itself, where one does wrong and no longer cares or even knows let alone feels guilty about committing evil.

This clearly corresponds to Kant’s last stage of depravity, where the maxim for good is subjected to that of evil entirely. The reason human beings fall into this state, Kant argues, is that we live in a world where we are always surrounded by incentives which compete with our commitment to prioritize our duty to the moral law. These incentives are generally directly linked to the question of our happiness, which need not but often does conflict with the moral law. According to Kant, we possess both incentives – to obtain happiness and to observe the moral law – by nature. We never lose our natural disposition to the moral law, even when we do evil, because otherwise we would never be able to do good again.24Kant, *Religion,* 6.46, trans. Pluhar/Palmquist, 52. By the same token, however, we always possess the capacity to do evil in virtue of free will.25Kant, *Religion,* 6.20, ‘Man nennt aber einen Menschen böse, nicht darum weil er Handlungen ausübt, welche böse (gesetzwidrig) sind; sondern weil diese so beschaffen sind, daß sie auf böse Maximen in ihm schließen lassen,’ trans. Pluhar/Palmquist, 20: ‘We call a human being evil, however, not because he performs actions that are evil (unlawful), but because they are so constituted as to allow one to infer evil maxims in him.’ See also Kant, *Religion,* 6.29, ‘Es ist aber hier nur vom Hange zum eigentlich, d. i. zum Moralisch=Bösen die Rede, welches, da es nur als Bestimmung der freien Willkür möglich ist, diese aber als gut oder böse nur durch ihre Maximen beurtheilt werden kann, in dem subjectiven Grunde der Möglichkeit der Abweichung der Maximen vom moralischen Gesetze bestehen muß und, wenn dieser Hang als allgemein zum Menschen (also als zum Charakter seiner Gattung) gehörig angenommen werden darf, ein natürlicher Hang des Menschen zum Bösen genannt werden wird,’ trans. Pluhar/Palmquist, 31: ‘Since this evil is possible only as the determination of the free power of choice, but this power can be judged good or evil only through its maxims, this evil must consist in the subjective basis for the possibility of the maxims’ deviation from the moral law; and if this propensity may be assumed to belong to the human being universally (and hence to belong to the character of his genus), it will be called a natural propensity of the human being to evil.’

This conclusion has significant ramifications for Kant’s account of the Christian doctrine of original sin. According to this doctrine, the human capacity for sinning or committing evil is the consequence of the original fall of a first man and woman through whom the propensity to evil became hereditary for all humans. As Kant insists, however, such a ‘temporal’ explanation for the origin of evil is unnecessary and irrelevant in light of a ‘rational’ one, which is rooted in an understanding of what is intrinsic to human nature and in this case of free will.26Kant, *Religion,* 6.40, ‘Von den freien Handlungen als solchen den Zeitursprung (gleich als von Naturwirkungen) zu suchen, ist also ein Widerspruch; mithin auch von der moralischen Beschaffenheit des Menschen, sofern sie als zufällig betrachtet wird, weil diese den Grund des Gebrauchs der Freiheit bedeutet, welcher (so wie der Bestimmungsgrund der freien Willkür überhaupt) lediglich in Vernunftvorstellungen gesucht werden muß,’ trans. Pluhar/Palmquist, 44: ‘To search for the temporal origin of free actions as free actions (just as for natural effects) is therefore a contradiction; and hence so is it to search for the temporal origin of the human being’s moral constitution insofar as this constitution is regarded as contingent, because moral constitution means the basis for the use of freedom, a basis which (like the determining basis of the free power of choice in general) must be sought solely in presentations of reason.’ For Kant, in other words, the fact that each human being is made by God to choose the good in virtue of a free will, which can nevertheless choose its opposite, evil, suffices to explain why each of us, on reaching the age of reason, inevitably realises this possibility. Although Kant acknowledges that there are many pressures on account of which we are inevitably bound to choose evil, these pressures do not *force* us to that end, which is always subject to free choice.27Kant, *Religion,* 6.41, ‘Eine jede böse Handlung muß, wenn man den Vernunftursprung derselben sucht, so betrachtet werden, als ob der Mensch unmittelbar aus dem Stande der Unschuld in sie gerathen wäre. Denn: wie auch sein voriges Verhalten gewesen sein mag, und welcherlei auch die auf ihn einfließenden Naturursachen sein mögen, imgleichen ob sie in oder außer ihm anzutreffen sind, so ist seine Handlung doch frei und durch keine dieser Ursachen bestimmt, kann also und muß immer als ein ursprünglicher Gebrauch seiner Willkür beurtheilt werden,’ trans. Pluhar/Palmquist, 45: ‘Every evil action must be regarded, when one seeks its rational origin, as if the human being had fallen into it directly from the state of innocence. For however his previous conduct may have been, and of whatever kind may be the natural causes influencing him, and likewise whether they are to be found within or outside him, his action is nonetheless free and not determined by any of these causes, and it therefore can and must always be judged as an original use of his power of choice.’ As Kant stresses, culpability for evil choices, and moral responsibility for choices overall, cannot be ascribed to us unless it is the case that the capacity for good and evil is written into the very power of free choice. Thus, Kant states:

What the human being is or is to become in the moral sense, good or evil, into that he must turn or have turned himself. Either must be an effect of his free power of choice; for otherwise it could not be imputed to him, and consequently he could not morally be either good or evil. When it is said, he is created good, then this can mean nothing more than this: he is created for the good, and the original predisposition in the human being is good. The human being himself is not yet good on that account; rather, according as he does or does not admit into his maxim the incentives contained in that predisposition (this must be left entirely to his free selection, he brings it about that he becomes good or evil).28Kant, *Religion within the Bounds,* 6.44, trans. Pluhar/Palmquist, 50.

Kant’s effective rejection of the doctrine of original sin represents a significant departure from the prior Christian tradition and even the orthodoxy of his own day. As I will aim to show in the rest of this paper, moreover, the related idea of ‘radical evil’, or a latent potential for evil in human nature was itself a significant movement away from the longstanding Western tradition, which had been anticipated already by certain medieval scholars who nevertheless still believed in the idea of original sin. To demonstrate this, I will outline briefly in the next section the contours of the tradition I have in mind, stemming from Augustine of Hippo and elaborated by Anselm of Canterbury, who affirmed that free will is capable only of choosing the good and denied that evil is a substantial option of the will. As I will show subsequently, this view was roundly rejected by early thirteenth-century Franciscans who offered a revisionist reading of Augustine and Anselm, which for the first time allowed that free will is able to vacillate between good and evil and affirmed that the possibility of doing so is essential to acquiring merit for good actions and punishment for evil ones.29*Magestri Alexandri de Hales Quaestiones disputatae ‘Antequam esset frater’*, vol. 1 (Quaracchi: Collegii S. Bonaventurae, 1960), qu. 33, dist. 3, memb. 4, 592–3. In affirming these things, the medieval Franciscan tradition anticipated the view that Kant later formulated and laid the groundwork for that development.

## Augustine on Evil and Free Will

Throughout his works, Augustine (354–430) famously argued that evil is not ‘something’ but is instead an absence or privation of the good.30Augustine, *Enchiridion* (*Faith, Hope, and Charity*), trans. Bernard M. Peebles, in *The Fathers of the Church* (Washington, D.C: The Catholic University of America Press, 1947; repr. 2002), 3.11, 376–77. His views in this regard stem from his belief that everything God created is good, and that existence itself is therefore a good. According to Augustine, evil deprives us of the goodness – or the forms of existence – that God intended for us. For instance, wounds and sickness deprive us of health; death removes life; and vices in the soul detract from the good of human nature. So construed, evil is parasitic on the good. While there can be good without evil, in other words, there can be no evil except in a being that is good.31Augustine, *Enchiridion*, trans. Peebles, 3.13, 378–79. As Augustine elaborates, however, natural beings, including human beings, are not immutably good like their creator. As temporal beings, they change and mature in their particular forms of goodness or existence, such as being human.32Augustine, *Enchiridion*, trans. Peebles, 12.4, 377–78.

The mutability of creaturely natures allowed for the possibility, which was realised by the first man and first woman, Adam and Eve, to fall away from the good and choose evil. As Augustine is quick to stress, however, free will was not strictly speaking the efficient or moving cause of this original sin. On his understanding, free will exists solely for the purpose of choosing the good and is incompatible with sin and evil.33Augustine, *On the Free Choice of the Will,* trans. Peter King (Cambridge: Cambridge University Press, 2010), 2.1, 30–31. At the fall, consequently, the will that failed to prefer the good served as what Augustine calls a ‘deficient’ cause of evil.34Augustine, *City of God, Books VIII-XVI* (*DCD*) 14.11, trans. Gerald G. Walsh and Grace Monahan, in *The Fathers of the Church* (Washington: D.C.: The Catholic University of America Press, 1952), 12.7, 257–58. As a result of this original sin, Augustine affirms that all human beings inherit the sin nature or tendency to sin, which each one actualises in their own way.

More specifically, original sin deprived human beings of the direct knowledge of God as the supreme being that was enjoyed by the first man and woman, limiting their contact to the temporal goods he has made. In consequence, Augustine notes that humans tend to confuse ‘this good and that good with *the* Good.’35Augustine, *De Trinitate (DT),* 8.8.12, trans. Stephen McKenna, in *The Fathers of the Church* (Washington: D.C.: The Catholic University of America Press, repr; 2002), 262–65. In other words, they stake hopes for happiness in finite and fleeting objects of direct human experience that cannot fully satisfy human desires. This does not mean that temporal things lack value for Augustine.36Augustine, *City of God,* 12.8, trans. Walsh/Monahan, 258–59; Augustine, *On the Free Choice,* 1.15, trans. King, 28–29. As we have seen, all created beings are good and exist for our benefit. The problem is simply that ascribing too much significance to them, or becoming dependent upon them for personal happiness, sets human beings up for disappointment and frustration in life.37Augustine, *On the Free Choice,* 1.11, trans. King, 20; 2.19, trans. King, 70–71.

In this context, belief in God as the supreme good is crucial, because it enables us to maintain a proper perspective on the things that are ‘not God’ and thus prevents us from developing unhealthy attachments to temporal things. That is precisely why Augustine insists that free will cannot rightly choose evil, namely, because choosing the lesser over the greater good – and thus choosing evil – means becoming chained or enslaved to things for the sake of our happiness which cannot guarantee it.38Augustine, *On Corruption and Grace,* 14.42, trans. King, 222. By contrast, the appropriate use of free will – to prefer the highest good or God above all else – makes it possible to appreciate and benefit from all things for what they are without becoming attached to them inappropriately.

## Anselm on Evil and Free Will

Anselm of Canterbury (1033–1109) was famous for advancing arguments from reason, without appealing to past authorities as the basis for his claims. Nevertheless, there are clear signs throughout his oeuvre of his reliance on Augustine, and in particular the latter’s understanding of the nature of free will. In his own work on the subject, *De libertate arbitrii,* Anselm states emphatically that ‘to be able to sin does not belong in the definition of free will. Furthermore, the power to sin is neither liberty nor a part of liberty.’39Anselm of Canterbury, *On Free Will*, in *The Major Works,* ed. G. R. Evans and Brian Davies (Oxford: Oxford University Press, 2008), 1, 176. For the Latin edition, see *S. Anselmi Cantuariensis Archiepiscopi Opera Omnia*, 2 vols, ed. Franciscus Salesius Schmitt (Stuttgart-Bad Cannstatt: Friedrich Frommann Verlag, 1968–84). As Anselm elaborates, ‘slavery is nothing other than the powerlessness not to sin.’40Anselm of Canterbury, *On Free Will*, 12, ed. Evans/Davies, 190. Thus, Anselm concludes that Adam and Eve ‘sinned through their own free will, though not insofar as it was free, that is, not through that thanks to which it was free and had the power not to sin or to serve sin, but rather by the power it had of sinning, unaided by its freedom not to sin or to be coerced into the servitude of sin.’41Anselm of Canterbury, *On Free Will*, 2, ed. Evans/Davies, 177.

As Anselm stresses, the will is only free when it seeks to ‘preserve rectitude for its own sake.’42Anselm of Canterbury, *On Free Will*, 3, ed. Evans/Davies, 179. In this regard, he echoes Augustine’s claim that ‘no one is happy but the righteous,’43Augustine, *City of God,* 14.25, trans. Walsh/Monahan, 404–5. that is, the person who is able to subordinate lower to higher goods and all goods to the knowledge that God is supreme. To do this is what Anselm following Augustine affirms that human beings fittingly ‘owe’ to God; this is how we fulfil his will for us. At the same time, preserving justice is in our own interests, since there is nothing about being a slave to sin that is consistent with human happiness and flourishing. As Augustine had affirmed, so Anselm posits that willing what God wants us to will, which is to will to treat him as the supreme good and regard all other goods as second to him, helps us put ordinary goods in proper perspective, so that desires for them do not become inordinate and enslaving. Thus, maintaining the just and proper order of our will, first to God, and then to other things, is what truly liberates the will. As Anselm therefore states, ‘there is nothing freer than a right will since no alien power can take away its rectitude.’44Anselm of Canterbury, *On Free Will*, 9, ed. Evans/Davies, 187.

## Evil in Early Franciscan Thought

Alexander of Hales (118–1245) is widely regarded as the founder of the medieval Franciscan intellectual tradition, even though he only became a friar at the age of fifty-six, that is, in 1236, after a long career teaching friars who attended his lectures at the University of Paris. Alexander was one of the most renowned and sophisticated theologians in this context, and many of the views he articulated in his Gloss on Lombard’s *Sentences* – a genre he himself established as the medieval equivalent of a doctoral thesis – eventually were adopted, albeit developed more fully, in the so-called *Summa Halensis* which was named for him and which he oversaw between 1236–45. This Summa, one of the first of its kind, was collaboratively authored by other members of the first-generation Franciscan school, especially Alexander’s chief collaborator, John of La Rochelle, who sought to articulate their own scholarly tradition for the first time. In his Gloss, Alexander lays the groundwork for the Summa’s attempt to answer the question whether evil exists, by distinguishing between multiple forms of being:

One must reply that ‘being’ has multiple meanings, for [there can be] natural being or moral being, and the latter has two senses, either graced or not. Therefore, one must say that if evil were a privation of a ‘being according to nature,’ [evil] is not that, which is lacking [in this instance]. Similarly, if there were a privation of a ‘moral being,’ [evil] is not that, which is lacking [in this instance]. However, if there were a privation of the ‘being of grace’ that indwells through the highest good, then [one] is deprived of the universal end, and in this sense it [evil] will be said not to exist.45*Magistri Alexandri de Hales Glossa in quatuor libros Sententiarum Petri Lombardi*, 4 vols, *In Librum Secundum* (Quaracchi, Florentiae: Collegii S. Bonaventurae, 1952), dist. 34, 327: ‘Dicendum quod ‘esse’ dicitur multipliciter. Aut enim esse naturae, aut esse moris; et hoc dupliciter, quoniam aut esse gratiae aut non. Dicendum ergo quod, si malum fuerit privatio ‘esse secundum naturam’, non est illud cuius est privatio. Similiter, si privetur ‘esse moris’, non est illud cuius est privatio. Si autem privetur ‘esse gratiae’ per summum bonum inhabitans, tunc privatur fine omnium, et sic dicetur ipsum non esse...Sed aliae privationes, ut caecitas et ignorantia, aliquo modo dicuntur esse, quoniam sunt poenae inflictae. Unde Augustinus, in libro *De libero arbitrio*: Mali poenae Deus auctor est, sed mali eulpae non.’

In this remarkable passage, Alexander basically rejects the longstanding privation theory of evil, which underpinned the Augustinian notion that free will is able only to choose the good. On his account, evil is not an absence of the good in the natural and moral contexts. In other words, it *does* exist, even if it involves a state of deficiency or lack, such as lack of virtue or lack of understanding. The only exception to this rule concerns the state of grace, where there is a genuine deprivation or privation of relationship to God in the person who lacks grace. In this case exclusively, evil is nothing.

In its second volume, which was redacted by an unknown author on the basis of works by Alexander and John, the *Summa Halensis* pays tribute to the long tradition of privation theory, quoting many of its famous proponents, including Augustine, Anselm, Pseudo-Dionysius, and Gregory the Great.46*Doctoris irrefragabilis Alexandri de Hales Ordinis minorum Summa theologica (SH)*, 4 vols, *Secunda Pars Secundi Libri* (Quaracchi, Florentiae: Collegii S. Bonaventurae, 1930), 2.2., In1, Tr1, Q1, 2. However, it lists a number of reasons why evil cannot be nothing but must in fact be something. For instance, the anonymous author argues that ‘every corruptive thing is active; but every active thing has a nature by which it acts. Therefore, every corruptive thing also has a nature; but evil is corruptive, because it corrupts the mode, the form and the order of a thing, as Augustine says and will be shown later. Therefore, it has in itself some nature by which it acts. Therefore, evil is something.’47*SH* 2.2., In1, Tr1, Q1, Contra a, 2: ‘Omne corruptivum est activum; sed omne activum habet natura per quam agit; ergo et omne corruptivum; sed malum corruptivum est, quia corrumpit modum, speciem et ordinem, ut dicit Augustinus et patebit infra ergo habet in se naturam aliquam per quam agit; ergo malum est aliquid.’

Furthermore, the Summa contends that evil must be something because it is subject to increase and decrease, and it contradicts the good.48*SH* 2.2., In1, Tr1, Q1, Contra c, 2: ‘Item, malum recipit magis et minus: aliquod enim malum est maius altero; ergo malum est aliquid. Contra d, 2: Item, malum pugnat contra bonum, ipsum expellendo; ergo malum est aliquid.’ The argument culminates in a passage where the Summa repeats and elaborates Alexander’s distinction between the two kinds of being, *esse naturae* and *esse moris*, to which it adds a third category of *esse rationis*, which had earlier been mentioned by Alexander in the context of treating Christ’s human nature*.*49*Magistri Alexandri de Hales Glossa in quatuor libros Sententiarum Petri Lombardi*, 4 vols, *In Librum Tertium* (Quaracchi, Florentiae: Collegii S. Bonaventurae, 1954), dist. 6, Respondeo, 82. In treating these categories, the Summa furthers Alexander’s thinking in rejecting the relevance of privation theory:

Being is said in many ways. There is rational being (*esse rationis*), according to which all things that have truth according to whatever mode, that is an adequation of the thing to the intellect, are called beings. According to this mode, evil exists because it deforms that in which it exists. There is furthermore natural being (*esse naturae*), and according to this mode, evil is said to be something, by reason of that which is subverted by malice, as an evil action by reason of being an action is said to be something. Finally there is moral being (*esse moris*), which is the being that preserves and retains the order of nature, as Boethius says in his book, *The Consolation of Philosophy*, according to which mode it is said that evil humans, in whom there is evil, lack being; according to this mode being is said as what is rightly ordered to the highest good, namely, God and so it is not evil.50Alexander of Hales, *Glossa in Librum Tertium*, Respondeo, 83: ‘Esse enim dicitur multipliciter. Est enim esse rationis, secundum quem modum quaecumque veritatem habent, id est adaequationem rei et intellectus, dicuntur entia: secundum hunc modum malitia est, cum deformat illud in quo est. Est etiam esse naturae: et secundum hunc modum, ratione eius quod substernitur malitiae, dicitur malum esse aliquid, ut mala actio ratione actionis dicitur esse aliquid. Est iterum esse moris, prout ‘esse est quod ordinem retinet servatque naturam’, et sic definit Boethius, in libro *De consolatione*, secundum quem modum dicuntur mali homines, in eo quod mali, non esse: secundum hunc modum dicitur esse, quod recte ordinatum est ad finem summum scilicet Deum, et sic malum non est*.*’

As this passage confirms, the Summist ascribes substantiality to evil in every case except the one that refers to the order human beings should have to God, namely, *esse moralis,* which has been lost as a result of sin and can only be restored through grace or a state of *esse gratia*. Thus, the Summa like Alexander distinguishes between *esse moralis* that is or is not subject to the redemptive power of grace. In another context, the Summa elaborates a related distinction between what it calls ‘primary being’ (*esse primum*) and ‘secondary being’ (*esse secundum*), which respectively correspond to *esse naturae*/*rationis* and *esse rationis*/*esse gratiae*.51*Doctoris irrefragabilis Alexandri de Hales Ordinis minorum Summa theologica (SH)*, 4 vols, *Liber Tertius* (Quaracchi, Florentiae: Collegii S. Bonaventurae, 1948), 3, P3, In1, Tr1, Q1, C1, Contra 5, 945: ‘Item duplex est opus divinum; creatio et recreatio; similiter est esse primum, scilicet esse naturae, et est esse secundum, scilicet bene esse.’ *SH* 3, P3, In1, Tr1, Q2, C1, Ar3, 961, 2: ‘Item, duplex est esse, scilicet esse primum et esse secundum: primum esse est esse naturae, secundum esse est bene esse sive esse ordinis; sed dignius est esse secundum qua primum; ergo perfectio secundi esse est dignior quam perfectio primi esse; sed perfectio primi esse est substantia; ergo et perfectio secundi esse est substantia; sed gratia est perfectio animae quantum ad secundum esse; ergo est substantia.’ As Strand notes, the Summa also speaks of primary being as the general *esse* or being of a human being as created by God, while secondary being concerns the well-being (*bene esse*) of the person and their re-creation after sin by God.

As Strand elaborates, the perfection of a human being in the primary sense occurs by virtue of the being’s own proper acts, such as its union with and enlivenment of the body.52See Vincent Strand, ‘The Ontology of Grace in Alexander of Hales and John of La Rochelle’, in *The Summa Halensis: Doctrines and Debates,* ed. Lydia Schumacher (Berlin: De Gruyter, 2020), 177 in 171–91. By contrast, the perfection of person in terms of their moral being requires an external act, that is, the infusion of grace.53*Doctoris irrefragabilis Alexandri de Hales Ordinis minorum Summa theologica (SH)*, 4 vols, *Liber Primus* (Quaracchi, Florentiae: Collegii S. Bonaventurae, 1924), 1, P1, In1, Tr2, Q3, T3, M2, C1, 79, contra a: ‘Differt esse et bene esse; sed prius est esse quam bene esse; ergo prius est perfici in esse quam in bene esse; sed anima quantum ad esse perficit corpus, gratia vero quantum ad bene esse; ergo necesse est prius animari carnem quam sit gratiae susceptiva; ergo Deus non potest inhabitare in carne per gratiam ante infusionem animae.’ This infusion is something special over and above the grace of natural being.54*SH* 1, P1, In1, Tr2, Q3, T3, M2, C1, Ad objecta 1, 77: ‘Unde licet modi generales non ponant aliquam qualitatem creatam, ponunt tamen aliquid creatum, sicut modus specialis, qui est inhabitare per gratiam.’ Thus, it can be lacking in a person who does not make themselves susceptible to grace through faith, that is, in one who has the capacity for *esse moris*, which is not realised through grace. As noted already, this is the sense that the Summa allows for a privation of being, namely, where a person fails to love God in the way that is key to moral well-being.

Augustine and other traditional privation theorists also affirmed that the lack of connection with God entails a privation of being. For these thinkers, however, this form of privation is linked to the others the Summa mentions, namely, the natural and the rational, which also entail a privation of the good. Thus, the Summa’s interpretation of privation effectively subverts the theory. The motives behind this re-reading are ones the Summa makes fairly clear in various places. First of all, affirming the legitimacy or substantiality of evil was a way to explain how God could incorporate evil into accomplishing his good purposes.55Oleg Bychkov, ‘Decor ex praesentia mali: Aesthetic Explanations of Evil in Thirteenth-Century Franciscan Thought’*, Recherches de théologie et philosophie médiévales* 68:1 (2001): 250–1. Furthermore, describing evil as a legitimate option for the will alongside the good was regarded as crucial to holding human beings responsible for their choices – to ascribing merit for good choices and demerit for evil ones.56*SH* 2.2, In1, Tr1, Q3, M2, Respondeo 1–2, 14. This brings us to the early Franciscan doctrine of free will.

## Free Will in Early Franciscan Thought

As in the discussion of evil, so in that of free will, Alexander of Hales was the pioneer of the position that became common currency in the early Franciscan school and the *Summa Halensis* specifically.57For further details, see Lydia Schumacher, ‘Free Choice’ in *Human Nature in Early Franciscan Thought: Philosophical Background and Theological Significance* (Cambridge: Cambridge University Press, 2023), 249–75. The fullest account of free will offered by Alexander is found not in his Gloss but in the Disputed Questions he wrote before he became a Franciscan friar in 1236, and specifically, question thirty-three. In this context, Alexander introduced a new authority on the topic alongside Augustine, Anselm and others, namely, John of Damascus, whose *De fide orthodoxa* had been translated into Latin from Greek in the mid-to-late twelfth century and became alongside others a major source for early thirteenth-century thinkers. In this work, the Damascene argues that every being that is generated or created from nothing is changeable.58Alexander of Hales, *Questiones disputatae* 33, disp. 1, memb. 1, contra 9, 569. On this basis, he argued that human beings specifically are able choose between different but equally legitimate good options.

Alexander interpreted the Damscene’s words as having a rather different meaning, however. In his view, the changeable nature of rational beings entails that they are able to preserve the good in which God created them or turn away from it in evil.59Alexander of Hales, *Questiones disputatae* 33, disp. 1, memb. 1, no. 16, 572. For the Franciscan, consequently, free will is flexible or able to choose between good and evil – a possibility that John of Damascus specifically rejected.60Michael Frede, ‘John of Damascus on Human Action, the Will, and Human Freedom,’ in *Byzantine Philosophy and its Ancient Sources*, ed. Katerina Ierodiakonou (Oxford: Clarendon Press, 2002), 63–95: Damascus ‘does not construe choice as inherently a choice between two [opposing] options, the good and the evil.’ Although Alexander acknowledges the views of both Augustine and Anselm, according to which free will is only able to choose the good, he argues that this refers to graced human nature, which is a kind of free will humans possess in common with God and good angels.61Alexander of Hales, *Questiones disputatae* 33, disp. 1, memb. 1, no. 20, 573. However human nature as such, not subject to grace, is not so ordered to the good but can alternate between good and evil, as noted above.62Ibid.: ‘Secundo modo libera voluntas in bono et in malo.’

Like the early Franciscan idea of evil, so this idea of free will was a significant revision of the traditional Augustinian view, which denied that evil could possess being or serve as a positive option for the will under any circumstances. Although Alexander paid lip service to the traditional Augustinian view that free choice consists in both reason and will – that is, both the judgement of reason and the execution of the judgment by the will, moreover, he ultimately argues that it is really more a matter of the will which follows through on reason’s decision.63Ibid., disp. 2, memb. 1, no. 51, 584. Riccardo Saccenti, *Conservare la retta volontà: L’atto morale nelle dottrine di Filippo il Cancelliere e Ugo di Saint-Cher (1225*–*1235)* (Bologna: Società editrice il Mulino, 2013). Similarly, sin consists primarily in the will in Alexander’s view, because we never really lose access to the knowledge of what is right to do, as Augustine believed occurred in virtue of the loss of the knowledge of God at the fall.64Alexander of Hales, *Questiones disputatae* 33, dist. 39, no. 4, 378. As hinted already, this prioritization of the will over the intellect was crucial in the Franciscan view for ascribing merit to human beings for their good and evil choices.

## The *Summa Halensis*

The *Summa Halensis* adopts and develops the views of Alexander of Hales on the nature of free choice in considerable detail.65*SH* 2.1, In4, Tr1, S2, Q3, T3, M1, 466–7. See translation of this section by Oleg Bychkov in *A Reader in Early Franciscan Theology: The Summa Halensis:* trans. Lydia Schumacher and Oleg Bychkov (New York: Fordham University Press, 2021), 228–47. Following Alexander, the Summa author invokes the Damascene’s argument at *De fide orthodoxa* 2.27 according to which creatures differ from God insofar they are brought into being from non-being and thus are subject to change. Although the Damascene inferred from this principle that free choice entails the ability ‘to be moved or not to be moved, to do or not to do, to desire or not to desire,’66*SH* 2.1, In4, Tr1, S2, Q3, Ti3, M3, C2, Ad 1, 480, citing *De fide orthodoxa* 2.26: ‘Liberi arbitrii est moveri vel non moveri, impetum facere et non facere, appetere et non appetere.’ any given thing, the Summist echoes Alexander in concluding that ‘free choice is that by which one is able to sin or do right.’67*SH* 2.1, In4, Tr1, S2, Q3, Ti3, M3, C5, Ad 2, 483: ‘Liberum arbitrium est quo homo potest peccare et recte agere.’ For the Summist, in summary, the human ability to choose does not involve a preference for this over that good but a vacillation between the opposites of good and evil.68*SH* 2.1, In4, Tr1, S2, Q3, T3, C3, Ar1, a, 475.

As the Summa writes: ‘free choice is what is able to choose or to refuse something. As, therefore, choice is indifferent to the two options, free choice is indifferent to good or evil.’69*SH* 2.1, In4, Tr1, S2, Q3, T3, C3, Ar1, a, 475: ‘Creatura rationalis vertibilis est secundum electionem, ex hoc enim liberum arbitrium est, quod eligere potest vel recusare. Cum ergo eligere sit indifferenter inter utrumque, et recusare similiter, ergo liberum arbitrium indifferenter dicitur boni et mali.’ In this regard, the Summa admits that sin is not a matter of free choice for Augustine and Anselm in their major works on free will. The author acknowledges that free choice is undermined by sin insofar as it entails slavery to unhealthy desires.70*SH* 2.1, In4, Tr1, S2, Q3, T3, C3, Ar1, Contra 4, 475, citing Augustine’s *De civitiate Dei* 14.11.1. However, he concludes that the servitude of sin is only incompatible with the freedom humans possess in a state of grace. When it comes to the state of nature, they possess the ability to do both good and evil.71*SH* 2.1, In4, Tr1, S2, Q3, T3, M3, C4, Ad objecta 2, 482. This is because human beings are created from nothing. As such, they are changeable and can therefore demit from the good in which they were created to do evil.72*SH* 2.2., In1, Tr1, Q3, M1, C3, Ad objecta 2, 11. They can do this by the power of free choice, which according to the Summa is the cause of both all the good as well as all the moral evil in the world.73*SH* 2.2., In1, Tr1, Q3, M1, C4, Respondeo, 13: ‘Liberum arbitrium est principium omnis mali culpabilis, si attendamus ad potentiam.’

This power, and in specific, the power to sin, involves inverting the proper order of what the Damascene called *thelesis* and *boulesis,* where the former concerns objects of sensory or intellectual desire, and the latter is ordered towards justice and ultimately God. The objects of *thelesis* are not intrinsically evil, for Alexander and the Summa authors, who build on John of La Rochelle’s account of these faculties in his *Summa de anima* of 1236 as well as the earlier.74Alexander of Hales also treats the *thelesis*/*boulesis* distinction in, *Questiones disputatae* 33, 590. Jean of La Rochelle, *Tractatus de divisione multiplici potentiarum animae: texte critique avec introduction, notes et tables*, ed. Pierre Michaud-Quantin (Paris: Vrin, 1964), 119: ‘Voluntatem autem diuidit in thelisim et bulisim, id est in voluntatem naturalem et rationalem; thelisis siue voluntas naturalis est respectu bonorum naturalium, que non possumus non appetere, sicut sunt esse, viuere, intelligere; voluntas rationalis est respectu bonorum non naturalium, que possumus velle et non velle.’ See also John of La Rochelle, *Summa de anima*, edited by Jacques Guy Bougerol (Paris: Vrin, 1995), 287: ‘Est enim bonum superius bonum racionale, quod dicitur honestum, et bonum simplex, quod sua ui nos trahit et sua dignitate nos allicit; et est bonum inferius bonum corporale delectabile carni, quod est bonum apparens siue secundum quid; et est bonum medium quod est bonum naturale quemadmodum esse et uiuere, et intelligere, et sentire, et quecumque sunt substancialia nature.’ Like Kant’s animal and human proclivities, however, they are determined and linked intrinsically to natural objects of human desire, which may not always be compatible with the purposes of justice and God’s will. By contrast, *boulesis* on John’s account, like Kant’s practical reason, frees it from temporal objects or matters of self-love so that it can prefer what is best in any given instance.

## Conclusion

The analysis above allows for drawing a number of parallels between the early Franciscan and Kantian positions on the nature of evil, not least in relation to free will. Whereas the longstanding Augustinian tradition had described evil as a privation or absence of the good, both early Franciscans and Kant attribute it a positive substance. This in the view of both is necessary to affirming that human beings are responsible for evil or immoral choices that it may make, namely, that they have the capacity for both good and evil equally. According to both Franciscans and Kant, this capacity is latent in free will, which has more to do with the choices of the will or practical reason, precisely because these choices are the final arbiter and indicator of our fundamental dispositions – the sources of merit or demerit for which we can be held accountable.

For both Kant and the early Franciscans, an evil choice involves inverting the order of the maxims that should be properly hierarchized, or putting considerations of personal happiness or self-love above what is right or just to do in any given instance. In the Franciscan view, as noted, what is right or just coincides with the will of God, whom human beings are supposedly commanded to love first and foremost.75See Richard Cross, ‘Natural Law, Moral Constructivism, and Duns Scotus’s Metaethics: The Centrality of Aesthetic Explanation,’ in *Reason, Religion and Natural Law: From Plato to Spinoza* (Oxford: Oxford University Press, 2012), 175–97. For Kant, by contrast, the rule of justice is inherent in reason itself and the maxim to ‘act as if the maxim of my action could become a universal law’. These are significant differences, which reflect the larger differences between the medieval era in which belief in God was taken for granted and the critical or Enlightenment era in which human autonomy from the divine was more strongly asserted. However, the differences to not detract from the fundamental similarities in perspective, which represent a broader shift, long in the making, away from the Augustinian idea that free will can only serve the good, that was founded on a privation theory of evil.

